# The impact of preoperative inflammatory indices on retinal vessel density and macular perfusion after anti-VEGF treatment in branch retinal vein occlusion (BRVO) using wide-field SS-OCTA

**DOI:** 10.3389/fmed.2026.1866632

**Published:** 2026-08-28

**Authors:** Rong Hu, Chen Zeng, Hao Cui, Ming Li, Chong Tang, Qi Li

**Affiliations:** 1 Department of Ophthalmology, The First Affiliated Hospital of Chongqing Medical University, Chongqing, China; 2 Department of Nursing, The First Affiliated Hospital of Chongqing Medical University, Chongqing, China

**Keywords:** intravitreal, macular edema, optical coherence tomography angiography, retinal vein occlusion, retinal vessel density

## Abstract

**Background:**

This study aimed to comprehensively explore the macular perfusion and topographic distributions of retinal vessel alterations in branch retinal vein occlusion (BRVO)-affected eyes that had been treated with an anti-vascular endothelial growth factor (anti-VEGF) intravitreal injection for 1 month prior, using swept-source optical coherence tomography angiography (SS-OCTA).

**Methods:**

Thirty BRVO-affected patients with macular edema (ME) who had received anti-VEGF treatment were included in this observational study. All participants were examined using wide-field SS–OCTA before and 1 month after intravitreal injection. The potential association between preoperative serological inflammatory indices and retinal blood flow was explored, and Changes in parameters before and after anti-vascular endothelial growth factor (anti-VEGF) treatment were analyzed.

**Results:**

After anti-VEGF treatment, central retinal thickness (CRT) was significantly reduced. The vessel density (VD) of nasal location (NL) in superficial retinal vessels (SRV), macular in choriocapillaris (CC), inferior-temporal location (ITL) and NL in SRV with large vessels removed were significant. The VD of macular and NL in in SRV with large vessels removed exhibited distinct interocular discrepancies between the affected eyes and the contralateral eyes. Serological parameters were closely associated with post-treatment ocular microvascular alterations.

**Conclusion:**

After intravitreous injection for one month, VD of certain retinal sectors of different retinal layers was significantly changed, especially in SRV, while distinctions in DRV were more easily spotted between the affected eyes and the contralateral eyes. Serological inflammatory indices may serve as potential biomarkers for postoperative alterations in VD.

## 1 Introduction

Retinal vein occlusion (RVO) is one of the most common retinal vascular diseases, with hypertension considered the most influential factor (1, 2). According to the site of the occlusion within the affected vessels, RVO is broadly classified into branch RVO (BRVO) and central RVO (CRVO), with BRVO occurring more frequently than CRVO. Approximately 10% of fellow eyes develop bilateral BRVO, compared to 5 to 6% at baseline, suggesting the potential impact of biological factors on the fellow eyes of patients with RVO (3).

Macular edema (ME) is a common complication in RVO, attributed to secondary leakage from retinal capillaries, which serves as an early pathological feature shown in some experimental animal models (4). In addition, occlusion of the cilio—retinal artery and whitening of the retina around the veins are associated with paracentral acute middle maculopathy (PAMM), a rare manifestation (5). Intravitreal anti-VEGF therapy has been established as the primary clinical modality for managing persistent vascular leakage and retinal edema. It exerts its efficacy by selectively inhibiting VEGF, the overexpression of which is driven by structural and functional impairment of the blood–retinal barrier (6). However, symptoms such as conjunctival hemorrhage and dry eye are most frequently reported after treatment, according to a large-scale retrospective observational study (7). Intravitreal anti-VEGF injections can reduce the risk of retinal ischemia and inhibit neovascularization in the retina and choroid; these therapeutic effects manifest as measurable changes in a panel of chorioretinal biomarkers, thereby stabilizing ocular biological function and maintaining fundus homeostasis (8, 9). Measurement of central retinal thickness (CRT) serves as an important imaging parameter in the diagnosis and management of macular edema (10). Inflammatory status is a critical modulator of RVO pathogenesis, particularly in young patients. This pathological process can be quantified via multiple inflammatory biomarkers, including prostaglandin E1 (PGE1) and prostaglandin E2 (PGE2) (11).

A variety of advanced imaging technologies enable the quantitative analysis of chorioretinal layer alterations secondary to these diseases (12, 13). Compared with fundus fluorescein angiography (FFA), optical coherence tomography angiography (OCTA) allows for non-invasive visualization of the retinal microvasculature. As a novel imaging modality based on sequential B-scanning, OCTA enables quantitative and qualitative evaluation of the retinal microvascular network. Furthermore, wide-field OCTA delivers highly reproducible, objective assessments of the peripheral retinal microstructure via high-resolution three-dimensional angiographic imaging (14). The extensive application of high-resolution ophthalmic imaging modalities has facilitated the detection of diverse chorioretinal microvascular alterations in patients with RVO (15, 16).

Nevertheless, previous studies have yielded controversial findings regarding the statistical differences in chorioretinal biomarkers before and after anti-VEGF treatment, as well as the interocular differences between affected and fellow eyes. In addition, few studies have investigated the association between baseline systemic inflammatory status and the therapeutic efficacy of intravitreal anti-VEGF injection. This investigation was designed as an exploratory retrospective study. Widefield swept-source OCTA (SS-OCTA) was employed to evaluate the impacts of anti-VEGF therapy on vascular density across distinct retinal layers and macular perfusion status. Furthermore, this study aimed to explore the potential association between baseline hematological parameters, inflammatory markers, and retinal blood flow.

## 2 Methods

### 2.1 Subjects

This retrospective observational study included 30 patients diagnosed with branch retinal vein occlusion (BRVO) accompanied by macular edema (ME) and treated with an intravitreal injection of anti-VEGF from July 2025 to December 2025 at the Department of Ophthalmology, The First Affiliated Hospital of Chongqing Medical University. All participants had posterior involvement and met the diagnostic criteria for unilateral BRVO with ME, as confirmed by slit-lamp biomicroscopy, fundus photography, macular optical coherence tomography (OCT), or fundus fluorescein angiography (FFA). All patients received a single intravitreal injection of conbercept (0.5 mg/0.05 mL) at baseline. Among these participants, retinal blood flow parameters were compared between the treated eye and the contralateral eye in 18 subjects.

This study was conducted in accordance with the Declaration of Helsinki and approved by the Institutional Review Board of The First Affiliated Hospital of Chongqing Medical University. Patients with a history of prior ocular surgery or concomitant ocular diseases, including age-related macular degeneration (AMD), polypoidal choroidal vasculopathy (PCV), and central serous chorioretinopathy (CSC), were excluded.

### 2.2 Data collection

Baseline demographic and clinical parameters, including age, sex, ocular laterality, and best corrected visual acuity (BCVA), were recorded. Baseline serological inflammatory indices were collected before treatment. Retinal microvascular parameters, retinal volume, central retinal thickness (CRT), and morphological characteristics of the foveal avascular zone (FAZ) were quantitatively measured before and after intravitreal anti-VEGF therapy.

### 2.3 OCTA imaging protocol

All participants underwent pupil dilation followed by swept-source OCTA (SS-OCTA; BMizar, TowardPi Medical Technology, Beijing, China) to acquire amplitude-decorrelation angiographic images. This system operates at 400,000 A-scans per second, utilizing a 1,060 nm swept-source vertical-cavity surface-emitting laser (VCSEL), with a scanning depth of 6 mm, an axial tissue resolution of 3.8 μm, a transverse resolution of 10 μm, and a maximum scanning field of 18 × 18 mm. Integrated with a 128 Hz ultra-high-frequency real-time eye-tracking module, built-in motion correction algorithms effectively minimized motion artifacts. The system automatically reconstructed 1280 cross-sectional images and calculated retinal vascular density (VD) via a high-order moment amplitude decorrelation angiography algorithm. Automated layer segmentation was applied to delineate the avascular retinal region and choroidal vascular layers.

**Figure 1:**
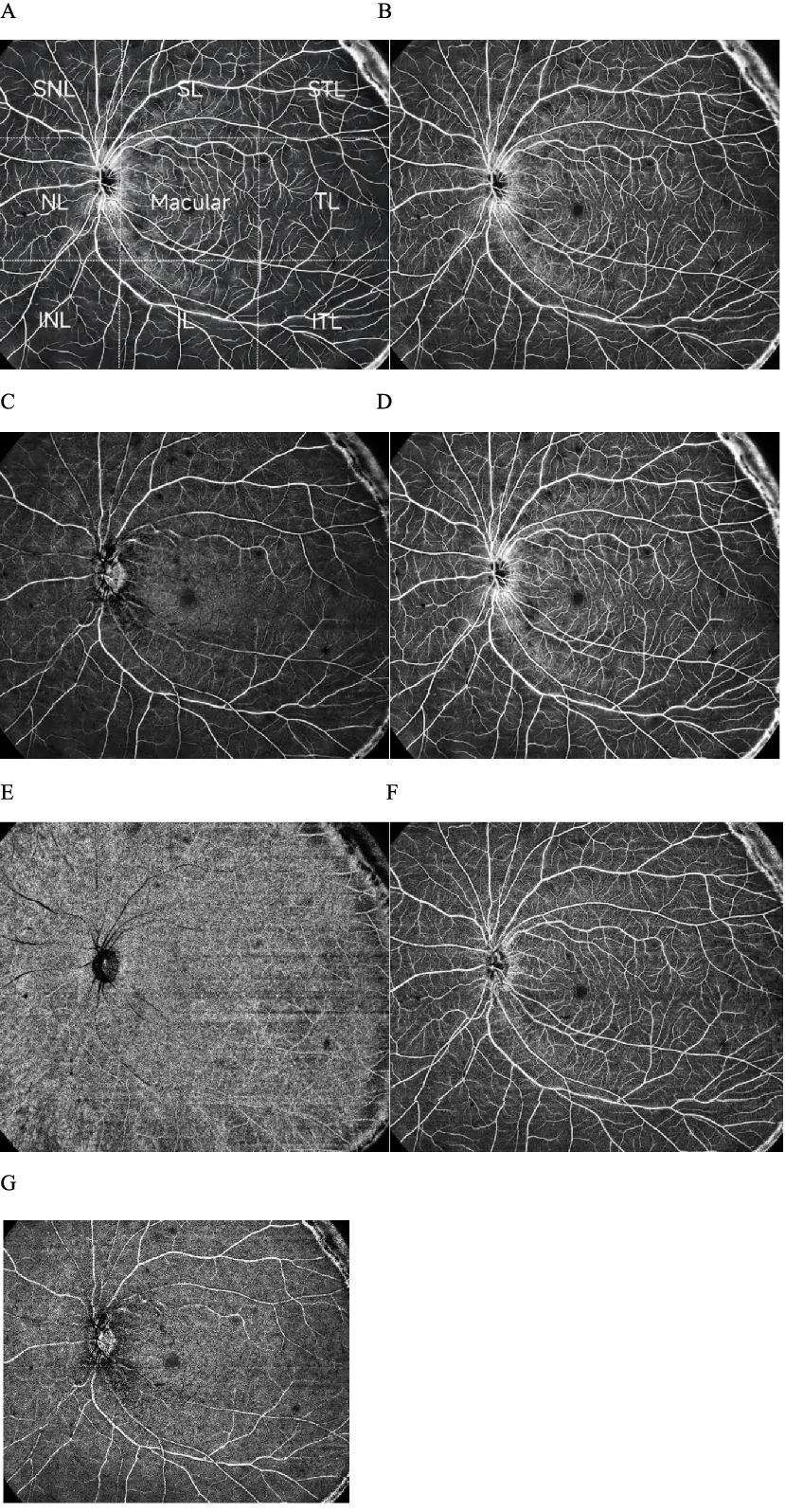
Various illustrations of retinal vascular layers and the choriocapillaris (CC). **(A)** Different anatomical sectors of the scan field, extending from the posterior pole to the peripheral retina. **(B)** Blood flow of SRV. **(C)** Blood flow of DRV. **(D)** Blood flow of the whole retina. **(E)** Blood flow of the CC. **(F)** Superficial vascular network of the retina. **(G)** Deep vascular network of the retina.

The 18 × 18 mm wide-field OCTA scan covered from the posterior pole to the peripheral retina, capturing an extensive fundus area that was segmented into four distinct layers: SRV, DRV, and CC, and large and medium-sized vessels of the choroid (LMVC). SRV was delineated from the internal limiting membrane (ILM) to 9 μm below the inner plexiform layer (IPL). The DRV was defined as the region from 6 μm below the IPL to 9 μm below the outer nuclear layer (ONL). An additional SRV en face angiogram with large vessels removed was generated by automatically excluding retinal vessels with a diameter ≥50 μm using the built-in algorithm of SS-OCTA. The CC corresponded to the area from Bruch’s membrane (BM) to 29 μm below the BM. Blood flow thermograms were captured to visually represent the blood flow in each retinal layer, with pseudocolor coding corresponding to VD value. The scan field was automatically divided into nine anatomic sectors: the central macula, superior (S), superotemporal (ST), temporal (T), inferior-temporal (IT), inferior (I), inferionasal (IN), nasal (N), and superonasal (SN). CRT were quantified across macular regions centered on the fovea with diameters of 1 mm and 3 mm respectively. The perimeter and the square of FAZ in separate retinal layers were collected (Figure 1).

### 2.4 Image quality control

Only images with a signal strength index (SSI) ≥ 7 were included in the analysis. Scans with significant motion artifacts, blink artifacts, or shadowing affecting >10% of the macular area were repeated at the time of acquisition. If image quality remained inadequate after three attempts, the eye was excluded. All scans were performed by the same experienced operator to minimize inter-operator variability.

### 2.5 Blood sample collection

Venous blood samples were collected from all patients within 1–3 days prior to anti-VEGF injection. A panel of hematological and inflammatory blood indices was measured, including white blood cell (WBC) count, platelet count, mean corpuscular hemoglobin concentration (MCHC), red blood cell (RBC) count, serum creatinine level, neutrophil-to-lymphocyte ratio (NLR), platelet-to-lymphocyte ratio (PLR), and systemic immune-inflammation index (SII).

### 2.6 Statistical analysis

Statistical analysis was performed using SPSS software (version 27.0.1, IBM Corp., Armonk, NY, USA). Continuous variables were expressed as the mean ± standard deviation (SD), or median (interquartile range, IQR), depending on distribution, whereas categorical variables were reported as frequencies and percentages ( n,% ). Data normality was verified using the Shapiro–Wilk test. Non-normally distributed parameters were analyzed using the Wilcoxon signed-rank test. Paired *t*-tests were performed to assess pre- versus 1-month postinjection changes in partitioned retinal parameters and interocular differences between affected and fellow eyes for normally distributed data. Cohen’s *d* was calculated to evaluate the effect size of pairwise comparisons defined as the mean difference divided by the pooled standard deviation. Effect size thresholds of 0.2, 0.5, and 0.8 were defined as small, medium, and large effects, respectively. Statistical significance was set at *p* < 0.05.

Spearman correlation analysis was performed to explore baseline associations between retinal microvascular indices and serological parameters. The Benjamini–Hochberg false discovery rate (FDR) correction was applied for multiple comparisons with a threshold of *q* = 0.05, and adjusted *q*-values were used to determine statistical significance (*p* < 0.05).

## 3 Results

### 3.1 General characteristics

A total of 30 patients with BRVO–ME were included in this retrospective study. The baseline characteristics are exhibited in Table 1. There were 14 males and 16 females who participated in the anti-VEGF treatment, and their average age was 60.30 ± 13.30 years. A series of baseline ocular measurements were specifically conducted on their individual characteristics of the retina (Table 1).

**Table 1:** Demographic and ocular characteristics of participants (*n* = 30).

Parameters	Total
No. of eyes	30
Age (years)	60.30 ± 13.30
Female (*n*)	16
Retinal thickness (μm)	294.67 ± 26.03
Retinal volume (μm^3^)	95.46 ± 8.44
Superficial FAZ area (μm^2^)	0.97 ± 1.59
Deep FAZ area (μm^2^)	2.11 ± 2.80
FAZ area (μm^2^)	0.92 ± 1.59
Superficial FAZ perimeter (μm)	4.38 ± 3.65
Deep FAZ perimeter (μm)	8.74 ± 6.56
FAZ perimeter (μm)	4.47 ± 3.62

### 3.2 Compartmental analyses of retinal blood flow indicators after treatment

As shown in Table 2, significant posttreatment changes in VD were observed across specific SRV regions, including the central macular area and global average retinal VD, regardless of large vessel involvement. No significant VD alterations were observed in the DRV layer. Additional analysis of CC demonstrated a significant reduction in macular sector VD following intravitreal injection.

**Table 2:** Compartmental analyses of retinal blood flow indicators after treatment (*n* = 30).

Retinal layers/sectors	Before	After	*p* value	Cohen’s d
SRV				
STL	34.57 ± 8.69	32.33 ± 8.18	0.318	0.186
TL	36.97 ± 5.79	34.10 ± 6.83	0.082	0.328
ITL	30.87 ± 8.92	27.10 ± 9.90	0.121	0.292
SL	38.60 ± 6.07	38.87 ± 8.22	0.874	0.029
Macular	36.17 ± 4.07	35.80 ± 4.27	0.739	0.061
IL	33.37 ± 9.36	32.37 ± 8.76	0.640	0.086
SNL	31.90 ± 8.42	28.43 ± 8.16	0.065	0.350
NL	36.03 ± 5.49	33.00 ± 6.55	0.031^*^	0.415
INL	27.40 ± 9.94	25.13 ± 9.01	0.356	0.171
DRV				
STL	38.13 ± 8.15	37.47 ± 6.55	0.745	0.060
TL	39.87 ± 4.42	39.13 ± 4.25	0.502	0.124
ITL	36.17 ± 6.85	33.93 ± 7.64	0.251	0.214
SL	40.07 ± 4.82	41.03 ± 5.98	0.482	0.130
Macular	37.47 ± 5.24	38.63 ± 4.86	0.357	0.171
IL	37.47 ± 6.05	37.37 ± 4.93	0.947	0.012
SNL	36.53 ± 8.69	34.07 ± 7.92	0.240	0.219
NL	38.83 ± 6.01	37.07 ± 6.22	0.252	0.213
INL	32.77 ± 8.78	31.10 ± 10.05	0.506	0.123
CC				
STL	46.73 ± 6.75	47.00 ± 6.80	0.891	0.025
TL	47.17 ± 2.95	47.40 ± 3.31	0.747	0.060
ITL	45.00 ± 4.84	45.07 ± 5.25	0.957	0.010
SL	47.00 ± 3.53	47.17 ± 5.79	0.891	0.025
Macular	42.00 ± 6.49	45.13 ± 3.16	0.006^**^	0.546
IL	44.87 ± 4.94	45.90 ± 4.05	0.391	0.159
SNL	44.50 ± 5.96	43.77 ± 8.08	0.704	0.070
NL	47.10 ± 4.72	46.87 ± 4.86	0.843	0.036
INL	40.00 ± 9.77	40.73 ± 9.50	0.778	0.052
SRV with large vessels removed				
STL	21.97 ± 6.31	19.97 ± 5.99	0.212	0.233
TL	23.60 ± 5.09	21.10 ± 5.33	0.085	0.326
ITL	19.97 ± 6.77	16.53 ± 6.65	0.044*	0.385
SL	24.83 ± 5.07	24.10 ± 5.58	0.552	0.110
Macular	25.53 ± 4.27	24.50 ± 4.16	0.362	0.169
IL	21.67 ± 6.36	19.87 ± 5.77	0.282	0.200
SNL	20.97 ± 6.64	18.43 ± 6.20	0.066	0.349
NL	23.67 ± 4.62	21.07 ± 5.09	0.030*	0.416
INL	18.27 ± 7.33	15.73 ± 6.30	0.158	0.265

*Refers to a *p*-value less than 0.05, reveals statistical significance. **Refers to a *p*-value less than 0.01.

Quantitative effect size analysis focusing on VD changes of SRV identified medium effect sizes (0.2 < Cohen’s *d* < 0.5) in NL, SNL, ITL, and TL sectors of the SRV, as well as in the overall average VD. After Benjamini–Hochberg FDR correction, only the macular sector of the choriocapillaris retained statistical significance.

### 3.3 Comparisons of retinal blood flow indicators between the affected eyes and the contralateral eyes after treatment

As retinal blood flow data were unavailable for some fellow eyes, comparisons of retinal blood flow parameters between affected eyes and contralateral eyes following treatment were conducted in a final cohort of 18 patients. No significant differences were found in VD of SRV and DRV. After excluding large retinal vessels, significant interocular differences in VD were observed in the macular and NL sectors between the affected eyes and the contralateral fellow eyes.

No significant changes were detected in average retinal VD following treatment (Table 3).

**Table 3:** Comparisons of retinal blood flow indicators between the affected eyes and the contralateral eyes after treatment (*n* = 18).

Retinal layers/sectors	The affected eye	The contralateral eye	*p* value
SRV			
STL	33.44 ± 6.62	34.61 ± 5.87	0.409
TL	35.5 ± 4.84	35.17 ± 5.96	0.855
ITL	26.61 ± 11.27	25.11 ± 8.78	0.659
SL	39.94 ± 7.0	37.5 ± 7.33	0.274
Macular	36.06 ± 3.54	37.5 ± 4.13	0.181
IL	33.0 ± 9.49	34.28 ± 8.07	0.666
SNL	27.0 ± 9.0	29.11 ± 9.91	0.508
NL	30.67 ± 7.6	35.56 ± 7.24	0.056
INL	25.06 ± 9.87	29.06 ± 7.63	0.183
DRV			
STL	39.94 ± 4.7	36.83 ± 6.47	0.115
TL	40.83 ± 4.33	39.44 ± 3.71	0.175
ITL	34.61 ± 8.96	30.61 ± 10.3	0.296
SL	41.56 ± 6.43	41.33 ± 3.85	0.505
Macular	40.33 ± 3.94	39.33 ± 3.61	0.293
IL	38.06 ± 7.54	36.72 ± 6.93	0.547
SNL	33.28 ± 8.19	34.78 ± 8.48	0.593
NL	37.22 ± 6.63	39.22 ± 5.72	0.535
INL	32.06 ± 9.6	33.11 ± 10.12	0.466
SRV with large vessels removed			
STL	21.39 ± 4.89	21.39 ± 4.98	1.000
TL	21.44 ± 4.44	22.56 ± 4.89	0.480
ITL	16.44 ± 5.9	15.11 ± 7.27	0.550
SL	23.28 ± 5.74	25.33 ± 4.65	0.246
Macular	23.94 ± 3.7	26.28 ± 2.65	0.037^*^
IL	20.17 ± 6.56	21.06 ± 4.66	0.642
SNL	16.28 ± 7.09	20.33 ± 7.01	0.094
NL	19.0 ± 6.33	23.78 ± 5.07	0.017^*^
INL	15.67 ± 6.26	18.89 ± 6.29	0.133
Retina			
STL	39.72 ± 4.9	37.11 ± 6.83	0.295
TL	40.17 ± 4.2	39.11 ± 4.07	0.287
ITL	33.56 ± 8.25	29.22 ± 10.84	0.235
SL	41.94 ± 6.34	42.72 ± 4.18	0.667
Macular	39.44 ± 3.47	39.28 ± 2.56	0.871
IL	38.44 ± 8.13	37.39 ± 7.4	0.634
SNL	32.72 ± 7.84	35.33 ± 7.98	0.329
NL	36.17 ± 6.8	39.72 ± 5.62	0.097
INL	31.89 ± 9.02	33.67 ± 9.7	0.341

*Refers to a *p*-value less than 0.05, reveals statistical significance. **Refers to a *p*-value less than 0.01.

### 3.4 Correlations between preoperative blood indicators and postoperative retinal vascular parameters

**Figure 2:**
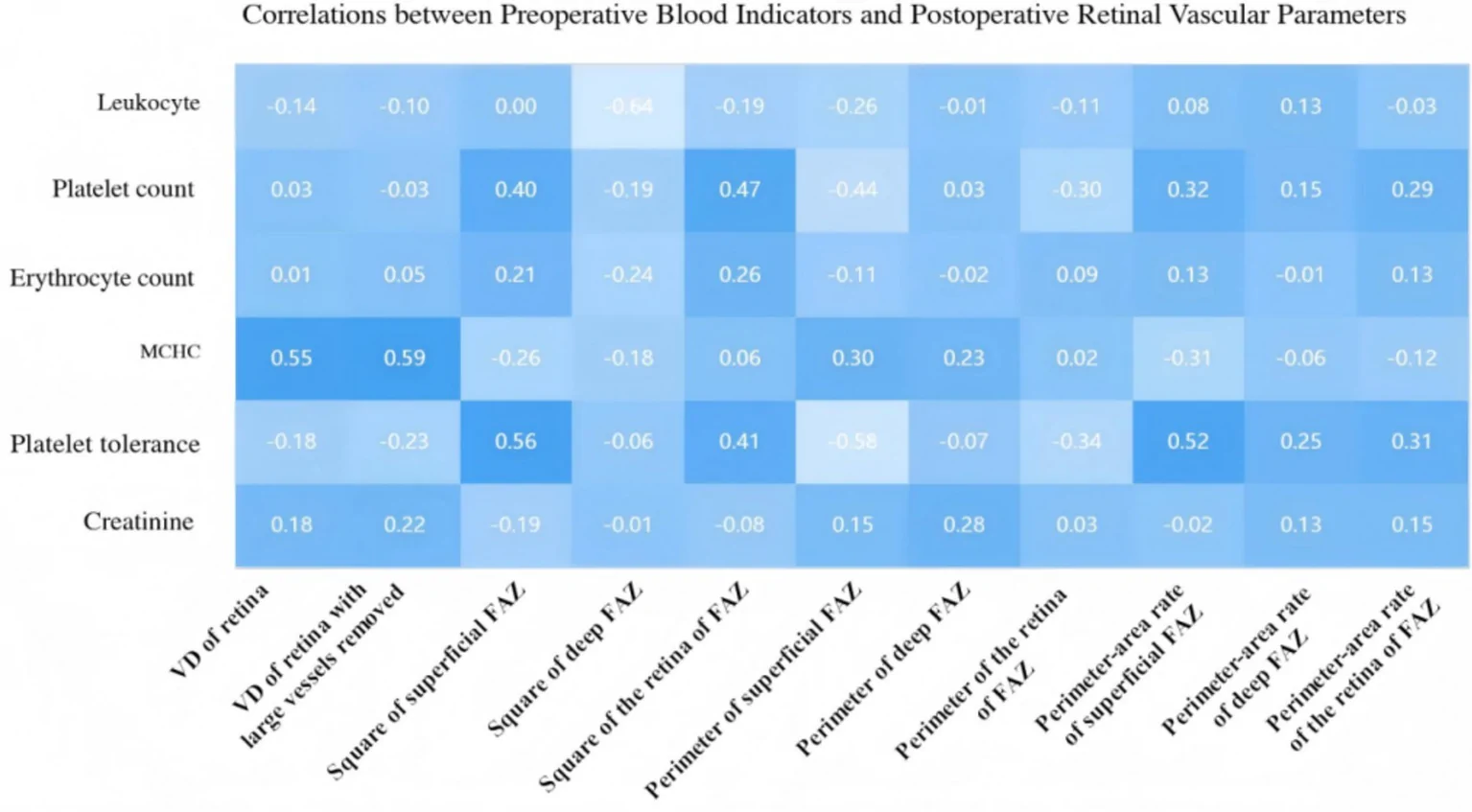
The correlation heatmap between preoperative blood indicators and postoperative retinal vascular parameters.

Spearman correlation was performed to quantify the potential association of various preoperative serological indices with postoperative microvascular alterations and macular perfusion, including leukocyte, plateletcrit, platelet count, and creatinine, shown in Table 4 and Figure 2. Regarding postoperative macular perfusion, the area of the superficial FAZ was significantly associated with baseline leukocyte level, plateletcrit, and platelet count. Both plateletcrit and platelet count were negatively correlated with postoperative changes in the superficial FAZ perimeter. MCHC was positively correlated with postoperative retinal VD. Creatinine and erythrocyte levels exerted no significant effects on postoperative retinal microvascular alterations (*p* > 0.05).

**Table 4:** Correlations between preoperative blood indicators and postoperative retinal vascular parameters.

		VD of retina	VD of retina with large vessels removed	Square of superficial FAZ	Square of deep FAZ	Square of the retina of FAZ	Perimeter of superficial FAZ	Perimeter of deep FAZ	Perimeter of the retina of FAZ	Perimeter-area rate of superficial FAZ	Perimeter-area rate of deep FAZ	Perimeter-area rate of the retina of FAZ
Leukocyte	*r*	−0.144	−0.102	0.001	−0.639^**^	−0.186	−0.262	−0.008	−0.110	0.082	0.127	−0.028
	*p*	0.484	0.620	0.996	0.000	0.362	0.196	0.970	0.591	0.690	0.538	0.893
Platelet count	*r*	0.034	−0.031	0.405*	−0.185	0.469*	−0.440*	0.026	−0.303	0.323	0.155	0.288
	*p*	0.870	0.879	0.040	0.365	0.016	0.024	0.901	0.133	0.108	0.451	0.154
Erythrocyte count	*r*	0.012	0.054	0.208	−0.241	0.257	−0.112	−0.020	0.087	0.126	−0.010	0.133
	*p*	0.953	0.793	0.307	0.236	0.206	0.586	0.925	0.673	0.541	0.962	0.518
MCHC	*r*	0.553^**^	0.594^**^	−0.265	−0.183	0.062	0.295	0.228	0.016	−0.313	−0.064	−0.120
	*p*	0.003	0.001	0.191	0.371	0.765	0.143	0.263	0.939	0.119	0.755	0.559
Platelet tolerance	*r*	−0.178	−0.233	0.565^**^	−0.058	0.408^**^	−0.583^**^	−0.068	−0.338	0.518^**^	0.250	0.311
	*p*	0.385	0.252	0.003	0.780	0.038	0.002	0.740	0.091	0.007	0.219	0.122
Creatinine	*r*	0.182	0.218	−0.188	−0.008	−0.082	0.146	0.280	0.034	−0.024	0.130	0.150
	*p*	0.385	0.295	0.369	0.972	0.697	0.486	0.175	0.871	0.908	0.537	0.474

*Refers to a *p*-value less than 0.05, revealing statistical significance. **Refers to a *p*-value less than 0.01.

### 3.5 Variations of central retinal thickness pre- and post-anti-VEGF therapy

Central retinal thickness (CRT) is defined as the average thickness of the retina between its inner and outer boundaries obtained within the 1 mm central macular circle. The inner retinal thickness (IRT), outer retinal thickness (ORT), and central retinal thickness (CRT) were quantified separately across macular regions centered on the fovea with diameters of 1 mm and 3 mm, respectively. Statistically significant reductions in IRT, ORT, and CRT were observed following anti-VEGF therapy (Table 5).

**Table 5:** Variations of central retinal thickness pre- and post-Anti-VEGF therapy (*n* = 30).

Retinal layers/sectors	Pre-	Post-	*p* value	Cohen’s *d*
1 × 1 mm				
IRT	97.10 ± 14.40	88.57 ± 13.71	0.0018^**^	0.497
ORT	197.53 ± 13.61	193.17 ± 17.15	0.0149^*^	0.237
CRT	294.67 ± 26.03	281.73 ± 28.55	0.0024^**^	0.386
3 × 3 mm				
IRT	86.20 ± 21.26	78.60 ± 17.11	0.0041^**^	0.315
ORT	187.03 ± 14.07	183.23 ± 15.41	0.0206^*^	0.236
CRT	270.53 ± 34.51	265.47 ± 28.37	0.0372^*^	0.150

*Refers to a *p*-value less than 0.05, reveals statistical significance. **Refers to a *p*-value less than 0.01.

## 4 Discussion

A series of remarkable findings were uncovered in this retrospective study. After intravitreal anti-VEGF injection, it is evident that a significant decrease in VD of the retina, while NL showed statistical distinction in SRV. VD of CC showed significant differences only in the macular region. No significant differences were detected in any individual sector of the DRV. Following further multiple comparison correction, only the macular sector of the CC exhibited significant differences. Distinctions were exhibited in IL in DRV between the injected eyes and their contralateral eyes, while the further quantitative analyses on the Cohen’s d value showed various differences in both the center macular and IL sectors in DRV with or without large vessels, as well as the average VD of the retina. According to correlation analyses, we also reported several potential relationships between preoperative ocular indicators and postoperative vascular parameters. For instance, platelet tolerance and platelet count were both suggested to be positively correlated with the square of the superficial FAZ, which was also negatively correlated with its perimeter. As for retinal vessel densities, the results indicated remarkable influences of individual MCHC on VD of SRV and the retina with or without large vessels after intravitreal injection, whereas no such significances were detected for other indicators. Owing to the negative results after comparing all *p*-values in the multiple correction analysis, we consider these statistical findings exploratory, unveiling certain potential pathological mechanisms, while requiring independent confirmation in adequately powered confirmatory studies.

In comparison with previous studies, our results demonstrated both similarities and differences. Consistent with Cheng’s report (14), we found a significant decrease in SRV density and a relatively stable DRV layer after anti-VEGF treatment. In another study, Sellam (17) reported a decrease in VD across all quadrants of both the superficial and deep layers except the fovea, which they attributed to perifoveal capillary disruption and resolution of intraretinal cysts. Ye (18), however, found a general increase in VD across both layers with a positive correlation to final visual acuity. These discrepancies may be partly explained by the distinct anatomy and physiology of the superficial and deep retinal plexuses. According to Ota (19), the superficial vessels are strongly related to macular edema, whereas the deep plexus is more closely linked to photoreceptor oxygen demands. Zuo gave insight into the outer retinal tubulation (ORT), a specific microstructural feature in the outer retina that was reflected as a circular or oval low-reflection space in B-scan (20, 21). Coscas (22) pointed out a more severe impact on the deep vascular layer in RVO, leading to downstream nonperfusion secondary to progressive leukocyte entrapment within retinal capillaries, and this phenomenon might be ameliorated following intravitreal injection. Ma (23) reported that an elevated IOP could also influence the macular and papillary vessel density observed by OCTA.

Based on current literature, the location of retinal vessels helps accurately mark leakage points while avoiding damage a laser spot could cause to vascular tissue (24). This regional predilection aligns with the findings of Noma (25), who reported that RVO-related alterations preferentially affect regions extending from the optic nerve to the nasal macula, where specific microstructures form part of the postreceptoral cone pathway. Cai (26) described significant reductions in retinal thickness across the macula and all quadrants, suggesting potential structural and functional recovery. Zhuang (27) noted the development of RNFL edema 1 week after intravitreal injection, which was secondary to a decreased VD and occurred predominantly in the nasal regions of the optic disc. Although there were several distinctions among current studies, we suggest that the nasal retina is particularly vulnerable to RVO-induced ischemia and shows the greatest response to anti-VEGF treatment.

We also considered why some alterations did not reach statistical significance. Santamaría (28) described permanent occlusion in areas devoid of blood flow that colocalized with cystoid spaces. While anti-VEGF treatment targets vascular abnormalities adjacent to cystic areas by reducing vessel diameter and microaneurysms, it cannot normalize VD in regions with established capillary loss. Other studies have suggested that a decrease in suspended scattering particles in motion (SSPiM) may also correlate with VD changes, reflecting reduced active leakage from a disrupted blood–retinal barrier (29, 30). These could serve as possible explanations and need confirmation in larger prospective studies.

This study showed that leukocyte, platelet count, MCHC and platelet tolerance before intravitreal anti-VEGF injection were associated with treatment response, including the morphology and hemodynamics of the retinal vessels. This is consistent with Noma (9), who reported that inflammatory cytokines can disrupt leukocyte function and slow retinal blood flow, thereby damaging the blood–retinal barrier and promoting macular edema. Niu (31) also linked higher neutrophil counts to increased vascular permeability via the release of MMP-9 and VEGF. In addition, elevated platelet counts may promote retinal vein thrombosis, and mean platelet volume (MPV) correlates with inflammation and thrombosis risk, thus contributing to RVO pathogenesis (32–36). MCHC was found to be significantly correlated with VD, possibly reflecting red blood cell stiffness and microvascular occlusion (37), while lower hemoglobin levels were associated with disruption of the ELM and EZ, predicting poor visual outcomes (38). Collectively, these exploratory findings suggest that systemic inflammatory and hematological markers may influence the biological response to anti-VEGF therapy in RVO, a hypothesis that warrants confirmation in larger prospective cohorts.

Accordingly, there was a list of attractive findings in our study. Given the alterations of VD in different chorioretinal parameters after treatment, we spot a significant decrease in certain regions in the superficial retinal layer, referring to the macular sector. The VD of NL and ITL in in SRV with large vessels removed exhibited distinct discrepancies before and after a single intravitreal anti-VEGF injection. Contrarily, comparing the affected eyes with the contralateral ones, distinctions were most prominent in SRV with large vessels removed, whereas negative in DRV. Significances were also explored in the correlation analysis, where the preoperative level of WBC was negatively associated with the square of the superficial FAZ postoperatively, while that of platelets was found to be positive. Several potential impacts were explored in the average level of hemoglobin in the meantime, whose increase would probably lead to more alterations in VD of the superficial and average retina. No significant correlations were observed between erythrocyte counts, creatinine levels and retinal vascular alterations as well as macular perfusion.

A series of limitations were retained in this study. First, it is an exploratory study that requires validation in larger cohorts in further studies, given the limited sample size and the short postoperation period that restricted the accuracy and universality of the conclusions. Besides, the limited number of participants may be insufficient to detect a minor effect size in several chorioretinal sectors, which were shown as restricted positive results in the Benjamini–Hochberg FDR correction. Therefore, a larger population and a longer postoperation period for further studies are definitely required. In addition, due to the retrospective design, the measurement results of contralateral eyes were incomplete for some patients, and no major systematic differences were observed between included and excluded patients based on available records. Further, since occlusion location for some patients with BRVO was not available in this retrospective cohort, the interpretability of sector-specific VD changes was therefore limited. In addition, although the study design was based on a broad vision field of 18 mm x 18 mm area, blood flow with a velocity of less than 0.3 mm/s in the retinal capillary was still missed in the detection area, nor was it in the microaneurysm (39). Given we did not directly assess several proposed pathophysiological mechanisms, including the presence of outer retinal tubulation, SSPiM and HRB, their potential roles in modulating VD changes remain speculative and require dedicated investigation in future studies. Besides, best-corrected visual acuity measurements for all enrolled patients at baseline (prior to injection) were collected. Regrettably, due to the retrospective design of this study, systematic collection of postoperative BCVA was not performed. Considering the absence of posttreatment parameters, such as visual acuity, in this retrospective study, the clinical significance of the observed sector-specific VD changes remains unclear, making it valuable for prospective studies to correlate VD alterations with their clinical outcomes.

## 5 Conclusion

In conclusion, the study deeply delved into the compartmental distinctions of various chorioretinal layers after intravitreal injection, referring to significant alterations of VD in specific sectors in the superficial retina and choroidal capillaries. VD of SRV with large vessels removed in the affected eyes compared to that in the contralateral eyes. A panel of potential correlations between several baseline serological parameters and postoperative retinal microvascular indices was also identified, which could serve as preliminary biomarkers for integration into future AI frameworks, reflecting the therapeutic effect of anti-VEGF intravitreal injection in the future (40, 41). However, the underlying pathological mechanisms and biomarkers for the treatment of BRVO–ME still require further investigation.

## Data Availability

The raw data supporting the conclusions of this article will be made available by the authors, without undue reservation.

## References

[1] Song P Xu Y Zha M Zhang Y Rudan I. Global epidemiology of retinal vein occlusion: a systematic review and meta-analysis of prevalence, incidence, and risk factors. J Glob Health. (2019) 9:010427. doi: 10.7189/jogh.09.010427, 31131101 PMC6513508

[2] O'Mahoney PR Wong DT Ray JG. Retinal vein occlusion and traditional risk factors for atherosclerosis. Arch Ophthalmol. (2008) 126:692–9. doi: 10.1001/archopht.126.5.692, 18474782

[3] Rogers SL RL McIntosh Lim L Mitchell P Cheung N Kowalski JW . Natural history of branch retinal vein occlusion: an evidence-based systematic review. Ophthalmology. (2010) 117:1094–1101.e5. doi: 10.1016/j.ophtha.2009.09.046 20430447

[4] Khayat M Lois N Williams M Stitt AW. Animal models of retinal vein occlusion. Invest Ophthalmol Vis Sci. (2017) 58:6175–92. doi: 10.1167/iovs.17-22713 29222552

[5] Lendzioszek M Bryl A Poppe E Zorena K Mrugacz M. Retinal vein occlusion-background knowledge and foreground knowledge prospects-a review. J Clin Med. (2024) 13:3950. doi: 10.3390/jcm13133950, 38999513 PMC11242360

[6] Munk MR Ceklic L Stillenmunkes R Chaudhary V Waheed N Chhablani J . Integrated assessment of OCT, multimodal imaging, and cytokine markers for predicting treatment responses in retinal vein occlusion associated macular edema: a comparative review of anti-VEGF and steroid therapies. Diagnostics (Basel). (2024) 14:1983. doi: 10.3390/diagnostics14171974 39272767 PMC11394301

[7] Plasencia C Eschle J Hatz K. Management and safety of same day bilateral intravitreal anti-VEGF injections in a treat-and-extend regimen. BMC Ophthalmol. (2025) 25:459. doi: 10.1186/s12886-025-03943-9 40814089 PMC12351889

[8] Wang JK. A review of randomized trials of approved pharmaceutical agents for macular edema secondary to retinal vein occlusion. Asia Pac J Ophthalmol (Phila). (2016) 5:159–64. doi: 10.1097/APO.0000000000000183, 26692257

[9] Noma H Yasuda K Shimura M. Cytokines and pathogenesis of central retinal vein occlusion. J Clin Med. (2020) 9:3457. doi: 10.3390/jcm9113610 33121094 PMC7692731

[10] Cao D Yao J Ting D Tan G. Artificial intelligence in predicting anti-VEGF treatment response in diabetic macular edema: current progress and future directions. Vis Neurosci. (2025) 42:e027. doi: 10.48130/vns-0025-0027

[11] Fong AC Schatz H. Central retinal vein occlusion in young adults. Surv Ophthalmol. (1993) 37:393–417. doi: 10.1016/0039-6257(93)90138-w, 8516752

[12] Chen N Zhu Z Yang W Wang Q. Progress in clinical research and applications of retinal vessel quantification technology based on fundus imaging. Front Bioeng Biotechnol. (2024) 12:1329263. doi: 10.3389/fbioe.2024.1329263, 38456011 PMC10917897

[13] Liu S Chen C Ning J Ding P Chen Y Xie T . Association between early inflammation and neurodegeneration in diabetic retinopathy: a narrative review. Vis Neurosci. (2025) 42:e012. doi: 10.48130/vns-0025-0012

[14] Cheng BT Mishra S Bryan JM Sadiq SA Sklar NC Suen EG . Retinal vessel density and treatment intensity among adults with retinal vein occlusion: a swept-source optical coherence tomography angiography study. J Clin Med. (2022) 11:2892. doi: 10.3390/jcm11102775 35629018 PMC9146926

[15] Suzuki N Hirano Y Tomiyasu T Esaki Y Uemura A Yasukawa T . Retinal hemodynamics seen on optical coherence tomography angiography before and after treatment of retinal vein occlusion. Invest Ophthalmol Vis Sci. (2016) 57:5681–7. doi: 10.1167/iovs.16-19706 27784073

[16] Hwang BE Kim M Park YH. Role of the choroidal vascularity index in branch retinal vein occlusion (BRVO) with macular edema. PLoS One. (2021) 16:e0258728. doi: 10.1371/journal.pone.0258728, 34673807 PMC8530297

[17] Sellam A Glacet-Bernard A Coscas F Miere A Coscas G Souied EH. Qualitative and quantitative follow-up using optical coherence tomography angiography of retinal vein occlusion treated with anti-VEGF. Retina. (2017) 37:1176–84. doi: 10.1097/IAE.0000000000001327, 27685676

[18] Ye P Zhu T Zheng F Zhou M Fang X Yao K. Microvascular comparison in younger and older patients with retinal vein occlusion analyzed by OCT angiography. BMC Ophthalmol. (2021) 21:161. doi: 10.1186/s12886-021-01946-w 33820544 PMC8022394

[19] Ota M Tsujikawa A Murakami T Kita M Miyamoto K Sakamoto A . Association between integrity of foveal photoreceptor layer and visual acuity in branch retinal vein occlusion. Br J Ophthalmol. (2007) 91:1644–9. doi: 10.1136/bjo.2007.118505 17504858 PMC2095528

[20] Zuo C Li X Zhang ZR Zhang MX. Characteristics and clinical significance of outer retinal tubulation in wet age-macular degeneration treated by anti-vascular endothelial growth factor through optical coherence tomography. Sichuan Da Xue Xue Bao Yi Xue Ban. (2016) 47:894–7. 28598120

[21] Al-Halafi AM. Outer retinal tubulation in diabetic macular edema following anti-VEGF treatment. Eye Vis (Lond). (2015) 2:9. doi: 10.1186/s40662-015-0020-5 26613090 PMC4660850

[22] Coscas F Glacet-Bernard A Miere A Caillaux V Uzzan J Lupidi M . Optical coherence tomography angiography in retinal vein occlusion: evaluation of superficial and deep capillary plexa. Am J Ophthalmol. (2016) 161:160–171.e2. doi: 10.1016/j.ajo.2015.10.008, 26476211

[23] Ma ZW Qiu WH Zhou DN Yang WH Pan XF Chen H. Changes in vessel density of the patients with narrow antenior chamber after an acute intraocular pressure elevation observed by OCT angiography. BMC Ophthalmol. (2019) 19:132. doi: 10.1186/s12886-019-1146-6, 31226955 PMC6588906

[24] Xu J Shen J Wan C Jiang Q Yan Z Yang W. A few-shot learning-based retinal vessel segmentation method for assisting in the central serous chorioretinopathy laser surgery. Front Med (Lausanne). (2022) 9:821565. doi: 10.3389/fmed.2022.821565, 35308538 PMC8927682

[25] Noma H Mimura T Kuse M Shimada K. Association of electroretinogram and morphological findings in central retinal vein occlusion with macular edema. Clin Ophthalmol. (2014) 8:191–7. doi: 10.2147/OPTH.S53979, 24531560 PMC3891666

[26] Cai L Wang J Huang D Cai Z. Effect of adjuvant conbercept on patients with macular edema induced by diabetic retinopathy and retinal vein occlusion undergoing laser treatment: impact on symptom improvement. Int J Gen Med. (2025) 18:6063–71. doi: 10.2147/IJGM.S479332 41078466 PMC12513388

[27] Zhuang X Su Y Li M Zhang L Mi L Ji Y . A prospective observation of influence of anti-VEGF on optic disc vasculature in nAMD patients. Photodiagn Photodyn Ther. (2024) 45:103863. doi: 10.1016/j.pdpdt.2023.103927 37890814

[28] Santamaría J Cobos E Biarnes M Caminal JM Rodriguez-Leor R Morwani R . Changes in vessel density patterns assessed with OCTA in patients with diabetic macular edema treated with anti-VEGF therapy. Acta Diabetol. (2024) 61:1385–92. doi: 10.1007/s00592-024-02298-5, 38802603 PMC11531438

[29] Kashani AH Green KM Kwon J Chu Z Zhang Q Wang RK . Suspended scattering particles in motion: a novel feature of OCT angiography in exudative maculopathies. Ophthalmol Retina. (2018) 2:694–702. doi: 10.1016/j.oret.2017.11.008 30221214 PMC6133252

[30] Ahn J Han S Ahn SM Kim SW Oh J. Clinical implications of suspended scattering particles in motion observed by optical coherence tomography angiography. Sci Rep. (2020) 10:15. doi: 10.1038/s41598-020-71952-5 31913306 PMC6949280

[31] Niu TT Xiao Y Lyu WJ Zhou H. Serologic parameters in young patients with retinal vein occlusion treated with anti-vascular endothelial growth factor. Int J Ophthalmol. (2025) 18:424–34. doi: 10.18240/ijo.2025.03.08, 40103956 PMC11865649

[32] Ieki Y Nishiwaki H Miura S Yamashiro K Nishijima K Nonaka A . Experimental macular edema induced by macular venule occlusion in monkey. Curr Eye Res. (2002) 25:123–31. doi: 10.1076/ceyr.25.2.123.13825 12525967

[33] Pinna A Porcu T Marzano J Boscia F Paliogiannis P Dore S . Mean platelet volume, red cell distribution width, and complete blood cell count indices in retinal vein occlusions. Ophthalmic Epidemiol. (2021) 28:39–47. doi: 10.1080/09286586.2020.1776338 32648802

[34] Liu Y Wang X Sheng Y Jin H Han L Xu J . Recurrence of macular edema in patients with branchretinal vein occlusion: a proteomic study. BMC Ophthalmol. (2024) 24:82. doi: 10.1186/s12886-024-03630-1 38388341 PMC10882909

[35] Fu H Qin B Hu Z Ma N Yang M Wei T . Neutrophil- and platelet-to-lymphocyte ratios are correlated with disease activity in rheumatoid arthritis. Clin Lab. (2015) 61:269–73. doi: 10.7754/clin.lab.2014.140822 25974992

[36] Karska-Basta I Kubicka-Trzaska A Pogrzebielski A Romanowska-Dixon B. A new insight into retinal vein occlusion pathogenesis. Klin Ocz. (2013) 115:269–74. 24908915

[37] Neuhouser AJ Sanders RN Konda M Uwaydat SH. Hemoglobin C trait presenting with bilateral central retinal vein occlusion. Retin Cases Brief Rep. (2023) 17:44–6. doi: 10.1097/ICB.0000000000001097, 33229918

[38] Gopalakrishnan N Nagaraju SP George NM Bhandary SV Kamath YS Shetty SS . Correlating the optical coherence tomography patterns and biomarkers of diabetic macular edema with hemoglobin level in diabetic kidney disease. Clin Ophthalmol. (2025) 19:209–15. doi: 10.2147/OPTH.S499285 39867346 PMC11762014

[39] Tokayer J Jia Y Dhalla AH Huang D. Blood flow velocity quantification using split-spectrum amplitude-decorrelation angiography with optical coherence tomography. Biomed Opt Express. (2013) 4:1909–24. doi: 10.1364/BOE.4.001909, 24156053 PMC3799655

[40] Li QR Rao LN Shen L Zheng YX Zhang QY Cui YX . Clinical investigation on the efficacy of vitrectomy in conjunction with anti-VEGF as opposed to anti-VEGF monotherapy in the management of PDR complicated with DME. Vis Neurosci. (2025) 42. doi: 10.1017/S0952523825000099

[41] Demir B Mishra A Gutierrez MPM Rasheed R Charitaki M Preston E . Outcomes of anti-VEGF treatment for macular edema secondary to central retinal vein occlusion in patients with poor baseline visual acuity. Can J Ophthalmol. (2024) 59:275–8. doi: 10.1016/j.jcjo.2023.07.004, 37517804

